# Increased Neointimal Thickening in Dystrophin-Deficient *mdx* Mice

**DOI:** 10.1371/journal.pone.0029904

**Published:** 2012-01-04

**Authors:** Uwe Rauch, Annelie Shami, Feng Zhang, Virginie Carmignac, Madeleine Durbeej, Anna Hultgårdh-Nilsson

**Affiliations:** Department of Experimental Medical Science, Lund University, Lund, Sweden; Universität Würzburg, Germany

## Abstract

**Background:**

The dystrophin gene, which is mutated in Duchenne muscular dystrophy (DMD), encodes a large cytoskeletal protein present in muscle fibers. While dystrophin in skeletal muscle has been extensively studied, the function of dystrophin in vascular smooth muscle is less clear. Here, we have analyzed the role of dystrophin in injury-induced arterial neointima formation.

**Methodology/Principal Findings:**

We detected a down-regulation of dystrophin, dystroglycan and β-sarcoglycan mRNA expression when vascular smooth muscle cells de-differentiate *in vitro*. To further mimic development of intimal lesions, we performed a collar-induced injury of the carotid artery in the *mdx* mouse, a model for DMD. As compared with control mice, *mdx* mice develop larger lesions with increased numbers of proliferating cells. *In vitro* experiments demonstrate increased migration of vascular smooth muscle cells from *mdx* mice whereas the rate of proliferation was similar in cells isolated from wild-type and *mdx* mice.

**Conclusions/Significance:**

These results show that dystrophin deficiency stimulates neointima formation and suggest that expression of dystrophin in vascular smooth muscle cells may protect the artery wall against injury-induced intimal thickening.

## Introduction

Duchenne muscular dystrophy (DMD) is a severe form of muscular dystrophy with X-linked recessive inheritance, caused by mutations in the gene encoding dystrophin [1]. DMD is characterized by progressive muscle wasting with a clinical onset at 2–5 years of age, ambulatory loss between ages 7 to 13 and death at 20–30 years of age due to cardiopulmonary failure [2]. The *mdx* (X-chromosome-linked muscular dystrophy) mouse is considered as the best animal model for DMD. Due to a point mutation in exon 23, *mdx* mice are missing dystrophin. Consequently, *mdx* mice develop muscular dystrophy, although the progressive muscle wasting presents itself in a much milder form than in humans, at least in the majority of the skeletal muscles. One notable exception is the *mdx* diaphragm, which reproduces the degenerative changes of muscular dystrophy. Yet, *mdx* mice have only a slightly shorter life-span compared to wild-type mice [3].

Dystrophin is a large intracellular protein that is localized to the sarcolemma through interactions with a large complex of membrane-associated and other cytosolic proteins, composed of dystroglycans (α, β), sarcoglycans (α, β, γ, δ), sarcospan and the syntrophins [4]–[6]. This dystrophin-glycoprotein complex (DGC) connects the subsarcolemmal cytoskeleton of a skeletal muscle fiber to its surrounding extracellular matrix and is believed to protect the skeletal muscle fiber from contraction-induced damage [7]–[9]. Dystrophin and the other components of the DGC are not only expressed in skeletal and cardiac muscle cells but also in vascular and other types of smooth muscle cells as well as in endothelial cells [10]–[15]. However, the functional role of dystrophin in vasculature is less clear. The presence of dystrophin in vascular smooth muscle appears to influence the nNOS-mediated attenuation of norepinephrine-mediated vasoconstriction that occurs in contracting muscles [16]. Moreover, carotid and mesenteric arteries from the *mdx* mouse model of dystrophin deficiency do not dilate properly under shear stress [12], [17]. Also, biomechanical properties of carotid arteries are altered in the *mdx* mice [17]. Finally, complete loss of the vascular smooth muscle DGC could contribute to the development of vascular spasm in sarcoglycan-deficient cardiomyopathy [18], [19] although more recent data suggest that cytokine release from degenerating cardiac myocytes may produce vascular spasm [20].

Vascular smooth muscle cells can undergo rapid and reversible phenotypic changes in response to stress and vascular injury. A differentiated vascular smooth muscle cell phenotype is characterized by expression of specific contractile and cytoskeletal proteins and the main function for this cell type is to regulate blood pressure and flow [21], . The other important function of the vascular smooth muscle cell is the repair mechanism, which is activated as a response to vascular injury. The smooth muscle cells then lose their contractility, start to proliferate and migrate into the innermost layer (intima) of the vessel, where they synthesize and deposit vast amounts of extracellular matrix (ECM) molecules [21], [22]. Such phenotypic modulation also takes place during the first week when primary smooth muscle cells are grown in culture [23], [24]. The vascular smooth muscle cell plays an important role both during the development of atherosclerotic plaques and in the formation of restenotic lesions. In the latter situation, endovascular procedures have an important limitation due to ECM production and aggressive smooth muscle cell proliferation, and much of current research is devoted to the prevention of these activities, for example by inhibition of smooth muscle cell matrix receptor interactions [25].

Here, we have analyzed the role of dystrophin in neointima formation as a response to vessel wall injury. For this purpose, we used a carotid periadventitial collar injury model. We found increased neointima formation in dystrophin-deficient *mdx* animals compared to control animals.

## Materials and Methods

### Animals

*Mdx* (C57BL/10ScSn-*mdx*/J) and control mice were obtained from Jackson Laboratory. All mouse experimentation was approved by the Malmö/Lund (Sweden) Ethical Committee for Animal Research (permit number M62-10). All mice were maintained in animal facilities according to animal care guidelines.

### Cell culture

Vascular smooth muscle cells from control and *mdx* mice (n = 6 of each) were isolated from aortas, which had been stripped of endothelial cells through scraping with a cotton swab. The tissue was digested in 0.3% collagenase (type II, Gibco) in Ham's F-12 medium (Gibco) supplemented with 50 µg/ml gentamicin, 5 mg/ml ascorbic acid (Sigma) and 1% bovine serum albumin and the cells were immediately seeded in plastic cell culture plates to be incubated at 37°C (5% CO_2_) in Ham's F-12 medium (Gibco) supplemented with 50 µg/ml gentamicin, 5 mg/ml ascorbic acid (Sigma) and 10% newborn calf serum (Gibco). *In vitro* proliferation assay was performed with the 5-Bromo-2′-deoxy-uridine (BrdU) Labelling and Detection Kit III (Roche). Vascular smooth muscle cells isolated from control and *mdx* mice were seeded (3500 per well in a minimum of 12 wells per parameter) in 96 well plates in F-12 medium containing 10% NCS (Gibco). BrdU labelling solution was added 6 hours after seeding. Cells were then incubated for 44 hours before quantification of incorporated BrdU was completed according to the manufacturer's instructions. For the *in vitro* transwell migration assay smooth muscle cells were seeded in the upper chamber of 8 micron transwells (Corning). F-12 medium in the upper chamber contained 1% bovine serum albumin (Sigma) and F-12 medium containing 10% NCS was added to the lower chamber. Cells were allowed to migrate for 20 hours. The filter was then cut from the chamber insert and cells that had migrated were counted (in area of 6 mm^2^ in the centre of the filter).

### RNA extraction, reverse transcription and quantitative real-time PCR

Total RNA was extracted from freshly isolated (contractile) or cultured (5–7 days; synthetic) smooth muscle cells from 6 and 8 wild type mice, respectively, using RNeasy mini kit (Qiagen). Complementary DNA was synthesized from l µg of total RNA with random primers and SuperScriptIII reverse transcriptase (Invitrogen) following manufacturer's instructions. Quantitative PCRs were performed in triplicate with the Maxima SYBR Green qPCR Master Mix (Fermentas). Expression of target and reference genes was monitored using a quantitative real-time RT-PCR method (Light Cycler, Roche) with primers for *dystrophin* (forward: 5′-AGCACAGGGCTATGAACAAAC-3′; reverse: 5′-ACTTCCGTCTCCATCAATGAAC-3′), *dystroglycan* (forward: 5′-AGAAAGTGGTAGAGAATGGGG-3′; reverse 5′-AGTAACAGGTGTAGGTGTGG-3′) and *β-sarcoglycan* (forward: 5′-AGCATGGAGTTCCACGAGAG-3′; reverse: 5′-GCTGGTGATGGAGGTCTTGT-3′) genes. Primers were designed using Primer3-Web (v. 0.4.0) (http://frodo.wi.mit.edu/primer3/input.htm). The amplification efficiency for each primer pair was evaluated by amplification of serially diluted template cDNAs (E = 10^-r/slope^). Efficiency corrected RNA levels (in arbitrary units) were calculated by using the formula E^-Ct^. Expression levels were then calculated relative to the endogenous control gene *GAPDH*.

### Periadventitial collar injury

At the age of approximately 4–5 months, mice (n = 11 for *mdx* and n = 6 for wild-type) were anesthetized with Ketamine (110 mg/kg)/Rompun (10–13 mg/kg) and the right carotid artery was carefully isolated under a dissecting microscope. A non-occlusive plastic collar was placed around the right carotid artery and the skin incision was closed, as described previously [26], [27]. Mice were sacrificed 21 days after collar placement and the carotid arteries were perfusion-fixed with Histochoice (Amresco), dissected out and stored in Histochoice at 4°C until paraffin embedded.

### Morphometric measurements

The carotid arteries were sectioned (5 µm) and one section every 100 µm was used for measurements of the atherosclerotic extent (approximately 10–20 sections per animal). Accustain elastic stain kit (Sigma) was used to visualize elastic laminae and areas of the different regions and circumferences were calculated using the Zeiss Axiovision image software (Zeiss). Lumen and medial (the area between the external elastic laminae and internal elastic laminae) areas were calculated. Lesion area was calculated by subtracting the lumen area from the internal elastic laminiae area.

### Immunohistochemistry

PCNA stainings of paraffin sections were performed using the PCNA staining kit from Invitrogen according to the manufacturer's instruction with the addition of quenching of endogenous peroxidase activity as well as heat induced antigen epitope retrieval (pH 6.0 for 20 minutes).

### Fluorescence immunohistochemistry

Aortas stored at 4°C were removed from Histochoice and cryoprotected in 30% sucrose phosphate buffer, embedded in OCT (Tissue-Tek OCT, Sakura Japan) compound for sectioning and frozen in isopentan/dry ice. Frozen tissue specimens were cut with a Microm HM 560 microtome into 6 µm sections and air dried on Superfrost plus slides (Menzel, Germany) for 30 minutes and stored at −80°C for further use. For immunofluorescence detection of antigens, frozen slides were left to dry at room temperature and submerged for 10 minutes in −20°C methanol, transferred to PBS and blocked with 5% goat serum in PBS for 1 hour at room temperature except stainings for smooth muscle α-actin and β-sarcoglycan, which were blocked with MOM-block, further processed following the basic protocol of the MOM-kit (Vector labs), and visualized with Cy3-streptavidin (Sigma). For laminin α2 chain, PCNA, and β-dystroglycan stainings the goat serum blocking solution was exchanged against antibodies diluted in blocking solution overnight. Slides were washed with PBS and incubated with Cy3-linked secondary antibody (goat anti rabbit Ig, Jackson) in PBS for 1 hour at room temperature, washed again and mounted with Vectashield (Vector labs). For the CD68 staining (performed together with PCNA) FITC- linked secondary antibodies (goat anti rat Ig, Jackson) were used. Images were taken on a Zeiss Axiophot 2 with a Hamamatsu C4742-95 camera and Openlab 5 software (Improvision). Antibodies against laminin α2 chain [28] were kindly provided by Dr. Lydia Sorokin, Muenster. Antibodies against β-dystroglycan were described previously [29]; smooth muscle α-actin antibodies (clone 1A4) were from Sigma; anti-β-sarcoglycan antibodies (clone βSarc/5B1) from Novocastra; anti-CD68 antibodies (clone FA-11) from AbD Serotec and anti-PCNA antiserum from Abcam (ab15497).

### Statistics

Results are expressed as mean ± SEM. For mRNA analyses, Student t-test was performed for analysis of significance and for the other measurements, the statistical significance between groups was determined by Mann-Whitney test (using GraphPad Prism version 4.0). *P*<0.05 was considered significant.

## Results

**Figure 1:**
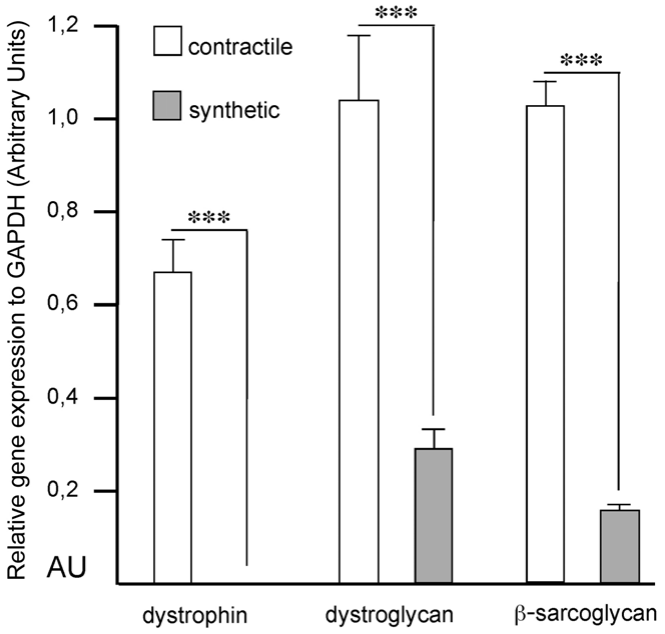
Reduction of dystrophin, dystroglycan and β-sarcoglycan mRNAs in synthetic smooth muscle cells. Relative amounts of dystrophin, dystroglycan and β-sarcoglycan mRNAs in contractile and synthetic smooth muscle cells (n = 6 and 8, respectively). The *GAPDH* gene expression served as a reference. ***, p<0.0001.

In response to vascular injury, medial smooth muscle cells lose their contractility and they migrate into the innermost layer of the vessel, where they start to proliferate and secrete ECM molecules. To investigate if dystrophin is altered when vascular smooth muscle cells undergo a phenotypic modulation from contractile to non-contractile synthetic cells, we used a well-established technique of culturing isolated mouse aortic vascular smooth muscle cells [23], [24]. Directly after collagenase digestion, when the smooth muscle cells are in a contractile phenotype, they strongly expressed dystrophin mRNA. After 5–7 days in culture, the cells had changed into a synthetic phenotype and at this time point the expression of dystrophin mRNA was shown to be dramatically decreased (Fig. 1). The reduction in dystrophin expression was accompanied by a significant reduction of dystroglycan and β-sarcoglycan mRNA expression (Fig. 1).

**Figure 2:**
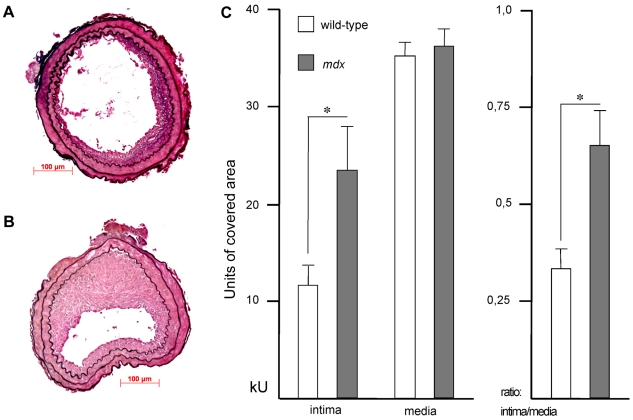
Increased neointima formation after vascular injury in *mdx* mice. Shown are representative sections of wild-type (A) and *mdx* (B) mice carotid arteries retrieved 3 weeks post injury and stained for elastin. Bar = 100 µm. Intimal (plaque) and medial area and intima/media ratio (C) was determined 21 days after vascular injury in wild-type (n = 6) and *mdx* (n = 10) mice. Data are expressed in thousands of units (kU) of covered area. *, p<0.05.

To study the role of dystrophin in neointima formation *in vivo*, we performed collar injury on carotid arteries in *mdx* and control mice. This type of vessel wall injury generates a lesion rich in smooth muscle cells and ECM, which is similar to human restenotic lesions [26], [30]. Histopathological examination of carotid artery cross section 21 days after injury revealed markedly enhanced neointimal thickening in *mdx* mice (Fig. 2B) compared with wild-type mice (Fig. 2A). To quantify the changes in vessel wall geometry, we measured the intimal and medial areas of *mdx* and control mice. In *mdx* mice, collar injury of the carotid artery caused a significant increase in neointimal area compared to wild-type animals (23 557±3409 units versus 11 771±1997 units, respectively, p<0.05, Fig. 2C), whereas there was no significant difference in the medial area between *mdx* and wild-type mice (36 193±1781 units versus 35 169±1393 units, respectively, p = NS, Fig. 2C). Hence, intima/media ratio remained significantly increased in *mdx* as compared with control mice (0.33±0.05 versus 0.65±0.09, p<0.05, Fig. 2C).

**Figure 3:**
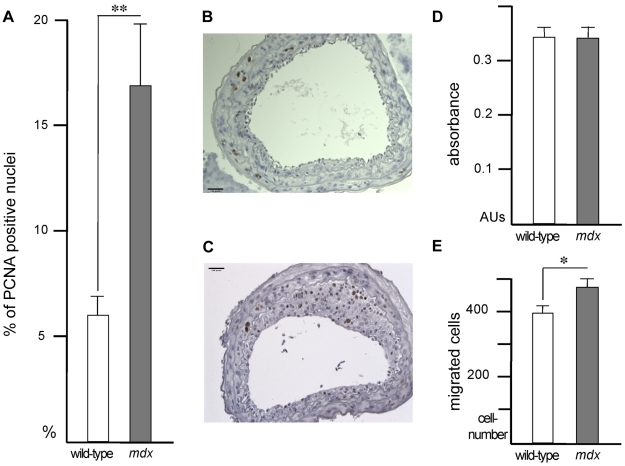
Assessment of cell proliferation in intima after vascular injury in *mdx* mice. The number of PCNA positive cells per total number of cells in intima (A) was determined 21 days after vascular injury in wild-type (n = 6) and *mdx* (n = 10) mice. **, p<0.01. Also shown are representative immunohistochemical sections of wild-type (B) and *mdx* (C) mice carotid arteries retrieved 3 weeks post injury and stained for PCNA. Bar = 32 µm. D and E present the results of *in vitro* studies with smooth muscle cells isolated from wild-type and *mdx* mice. D: Rate of BrdU incorporation during 2 days in culture determined by measurement of the absorbance of peroxidase-modified ABTS substrate (AUs: absorbance units). E: Number of cells, which migrated towards serum-containing medium, observed per 6 mm^2^ of the filter bottom.

We also assessed the rate of proliferation of vascular smooth muscle cells in the media and neointima using an antibody against the proliferative marker PCNA. The mean number of PCNA-positive cells was approximately 2-fold higher in the neointimal lesions of *mdx* mice than those of wild-type mice (16.9±2.9% versus 6.0±0.9%, respectively, p = 0.0013, Fig. 3A–C), whereas there was no significant difference between the two groups regarding the number of PCNA-positive cells in the media (data not shown). It is well documented that a majority of the cells in a mechanically induced arterial lesion at a late time point of 21 days are vascular smooth muscle cells [31]. To further demonstrate that most PCNA-positive cells were vascular smooth muscle cells, rather than inflammatory cells, we performed double immunofluorescence staining using antibodies against PCNA and the macrophage/leukocyte marker CD68. Indeed, CD68-positive cells were typically localized in the media and adventitia, whereas PCNA-positive cells were located in the neointima of injured arteries from wild-type and *mdx* mice (Fig. S1). A few CD68-positive cells, which also were positive for PCNA, were found in the neointima of injured arteries from *mdx* mice. Nevertheless, a majority of the PCNA-positive cells are likely to be vascular smooth muscle cells.

We next evaluated proliferation as well as migration of vascular smooth muscle cells *in vitro*. We could not detect any difference the rate of proliferation between vascular smooth muscle cells isolated from wild-type and *mdx* mice (Fig. 3D) which may be a consequence of the rapid down regulation of dystrophin mRNA in cultured wild-type vascular smooth muscle cells (Fig. 1). However, the serum-induced migration was shown to be increased with around 20% (476 cells/6 mm^2^±23 versus 396±15 cells/6 mm^2^, p<0.01) in vascular smooth muscle cells from *mdx* mice (Fig. 3E). In line with these findings, differential responses of smooth muscle cells with respect to proliferation and migration *in vitro* have been observed previously [32]–[34]. In addition, permanent adaptive molecular changes during development of *mdx* smooth muscle to compensate for the lack of dystrophin may enable the cells to migrate, but not proliferate more efficiently *in vitro*. Finally, we cannot completely exclude the possibility that the *in vivo* defects are due to loss of dystrophin expression in other cell types (e.g. endothelial cells) rather than loss of dystrophin in vascular smooth muscle cells.

**Figure 4:**
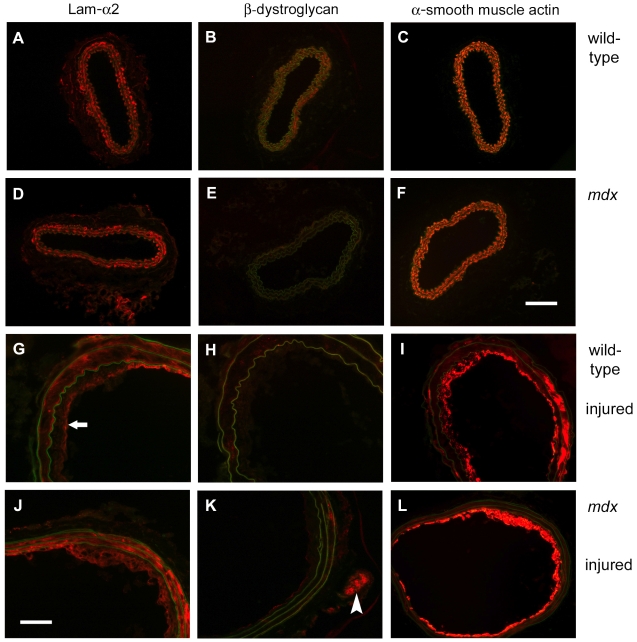
Immunohistochemical staining of laminin α2 chain, β-dystroglycan, and smooth muscle α-actin in uninjured and injured carotid arteries. Carotid arteries of uninjured wild-type (A–C) and *mdx* (D–F) mice and of injured wild-type (G–I) and *mdx* (J–L) were stained with anti-sera against laminin α2 chain (A, D, G, J) and β-dystroglycan (B, E, H, K) and an antibody against smooth muscle α-actin (C, F, I, L). Autofluorescence of the tissue detected in the green channel, in particular of the elastin layers of the media, is presented in green. Note, that the arrow (in G) is pointing out the luminal border of the plaque, while the arrowhead (in K) is pointing out a β-dystroglycan positive peripheral nerve. Size bars represent 100 µm for A-F, I & L and 50 µm for G, H, J & K.

Dystrophin is anchored to the sarcolemma through interactions with dystroglycan, which in turn binds to laminin α2 chain, the major laminin α chain in the basement membrane covering skeletal muscle. Also, sarcoglycans are tightly associated with dystroglycan [35], [36]. To determine whether expression of dystroglycan, laminin α2 chain and β-sarcoglycan is altered during neointima formation, we performed immunofluorescent analyses. The appearance of laminin α2 chain between the elastin layers within the media, demonstrating the presence of a basement membrane like structures around each individual smooth muscle cell [37], [38], was similar in uninjured carotid vessels of wild-type and *mdx* mice (Fig. 4). In these uninjured vessels it coincided with smooth muscle α-actin, which was present within the cells also located between the elastic layers of the media (Fig. 4). β-dystroglycan was evenly and consistently observed in the media of wild-type mice but less consistently observed in the media of *mdx* mice (Fig. 4). β-sarcoglycan, on the other hand, was expressed in the media of both uninjured wild-type and *mdx* vessels (Fig. S2).

In both wild-type and *mdx* mice, laminin α2 staining was present in the media of injured arteries. In addition, in wild-type mice laminin α2 chain was also lining the luminal border of the neointima, as observed previously [38], while in *mdx* mice laminin α2 chain was observed throughout the neointima (Fig. 4). In injured vessels, β-dystroglycan was generally only weakly and inconsistently observed in the media and neointima of both wild-type and *mdx* mice (Fig. 4). In injured arteries, smooth muscle α-actin was mostly observed in the neointima (Fig. 4). Finally, a weaker β-sarcoglycan staining was observed in injured wild-type and *mdx* arteries (Fig. S2).

## Discussion

This is the first study to evaluate the role of dystrophin in neointima formation as a response to mechanical injury. Dystrophin is absent in DMD or reduced or truncated in the milder variant Becker muscular dystrophy. These two disorders are characterized by skeletal muscle weakness and cardiomyopathy [2]. We found that neointima formation after collar-injury is increased in dystrophin-deficient animals. Hence, it could be that patients with dystrophin deficiency may more easily develop atherosclerotic lesions as well as restenotic lesions as a response to angioplasty. While atherosclerosis may not be a concern for juvenile DMD males it should perhaps be taken into account for the clinical care of older DMD men. Nevertheless, it is yet to be determined whether DMD patients are more susceptible to development of atherosclerotic and restenotic lesions.

Like it has already been pointed out, upon activation, the vascular smooth muscle cells lose their contractility, and migrate from the media into the intima, where they synthesize and deposit large amounts of ECM proteins. This makes the smooth muscle cell an important player for the fate of both the primary atherosclerotic lesion as well as for a restenotic lesion. In the primary lesion, the smooth muscle cells direct the formation and quality of the fibrous cap covering the atherosclerotic tissue and protect it from being exposed to the blood [39]. Rupture of a weak fibrous cap induces a rapid thrombus formation with subsequent myocardial infarction and stroke. On the other hand, the repair process by the smooth muscle cell as a response to endovascular procedure may become uncontrolled, leading to massive proliferation and matrix production, a situation that may result in formation of restenotic lesions [30].

In uninjured carotid vessels the individual smooth muscle cells within the media are individually encased by basement membrane like structures containing laminin α2 chain [37], [38]. While in wild-type animals the actin cytoskeleton can be linked to the basement membrane via the DGC and alternatively via integrins, only the latter interaction may be functional in *mdx* mice. After injury, a co-distribution of smooth muscle α-actin and laminin α2 chain staining is no longer evident, since activated cells leave their native environment and migrate into the neointima, where they can switch their integrin receptor repertoire to other subtypes binding fibronectin, collagen, and other laminin chains. The reduction of dystroglycan staining indicates that cytoskeletal ECM interactions mediated by the DGC become less important or are even inhibitory for this process.

Interestingly, perlecan, a basement membrane resident heparan sulfate proteoglycan, which binds via its core protein in a similar way as laminin α2 chain to the dystroglycan complex, has been implicated in the atherosclerosis process, since it is reduced in human carotid atherosclerotic lesions [40]. Here, it is concentrated in a similar way as laminin α2 chain along the luminal border [40]. This leaves smooth muscle cells located within the inner neointima without apparent extracellular ligand for the DGC. Furthermore, these observations and our *in vitro* data are in accordance with the results from Quignard et al. demonstrating lack of dystrophin in neointimal cells two weeks after mechanical injury. [41]. Notably, transgenic mice with a targeted deletion of the major attachment sites for glycosaminoglycan chains in the perlecan core protein, which is unlikely to interfere with its ability to bind dystroglycan, display similar to *mdx* mice upon mechanical injury increased intimal hyperplasia and smooth muscle cell proliferation [40]. Hence, it is likely that dystrophin, just like perlecan, plays an important role in the restenotic process. It will now be interesting to determine whether dystroglycan, connecting these two molecules, also is involved in neointima formation as a response to vessel wall injury and whether the DGC plays a role in the development of atherosclerotic lesions.

## Supporting Information

Figure S1 **Immunofluorescence staining of PCNA and CD68 positive macrophages in injured carotid arteries.** Sections of carotid lesions from wild-type (A, B, C) and *mdx* (D, E, F) mice were stained for PCNA (A and D) and CD68 (B and E). Images from these staining were merged in C and F showing that PCNA-positive cells were located in the neointima where few CD68-positive cells were found. Bar = 50 µm. The elastic laminae are shown by autofluorescence in the green channel.
(TIFF)

Figure S2 **Immunohistochemical staining of β-sarcoglycan in uninjured and injured carotid arteries.** Carotid arteries of uninjured wild-type (A, E) and *mdx* (B, F) mice and of injured wild-type (C, G) and *mdx* (D, H) were stained with a primary antibody against β-sarcoglycan (A–D) or without (E–H, control for anti-mouse IgG) (in red). Autofluorescence, in particular of the elastin layers of the media, is presented in green. Note the background staining of the secondary anti-mouse IgGs in the control of the injured, but not uninjured media. Bar = 100 µm.
(TIFF)

## References

[1] Hoffman EP, Brown RJ, Kunkel LM (1987). Dystrophin: the protein product of the Duchenne muscular dystrophy locus.. Cell.

[2] Engel AG, Ozawa E, Engel AG, Franzini-Armstrong C (2004). Dystrophinopathies. Myology..

[3] Willmann R, Possekel S, Dubach-Powell J, Meier T, Rüegg MA (2009). Mammalian animal models for Duchenne muscular dystrophy.. Neuromuscul Disord.

[4] Campbell KP, Kahl SD (1989). Association of dystrophin and an integral membrane glycoprotein.. Nature.

[5] Ervasti JM, Ohlendieck K, Kahl SD, Campbell KP (1990). Deficiency of a glycoprotein component of the dystrophin complex in dystrophic muscle.. Nature.

[6] Yoshida M, Ozawa E (1990). Glycoprotein complex anchoring dystrophin to sarcolemma.. J Biochem.

[7] Ibraghimov-Beskrovnaya O, Ervasti JM, Leveille CJ, Slaughter CA, Sernett SW (1992). Primary structure of dystrophin-associated glycoproteins linking dystrophin to the extracellular matrix.. Nature.

[8] Ervasti JM, Campbell KP (1993). A role for dystrophin associated glycoproteins as a transmembrane linker between laminin and actin.. J Cell Biol.

[9] Petrof BJ, Shrager JB, Stedman HH, Kelly AM, Sweeney HL (1993). Dystrophin protects the sarcolemma from stresses developed during muscle contraction.. Proc Natl Acad Sci USA.

[10] Houzelstein D, Lyons GE, Chamberlain J, Buckingham ME (1992). Localization of dystrophin gene transcripts during mouse embryogenesis.. J Cell Biol.

[11] Harricane MC, Fabbrizio E, Lees D, Prades C, Travo P (1994). Dystrophin does not influence regular cytoskeletal architecture but is required for contractile performance in smooth muscle aortic cells.. Cell Biol Int.

[12] Loufrani L, Matrougui K, Gorny D, Duriez M, Blanc I (2001). Flow (shear stress)-induced endothelium-dependent dilation is altered in mice lacking the gene encoding for dystrophin.. Circulation.

[13] Straub V, Ettinger AJ, Durbeej M, Venzke DP, Cutshall S (1999). ε-sarcoglycan replaces α-sarcoglycan in smooth muscle to form a unique dystrophin-glycoprotein complex.. J Biol Chem.

[14] Barresi R, Moore SA, Stolle CA, Mendell JR, Campbell KP (2000). Expression of γ-sarcoglycan in smooth muscle and its interaction with the smooth muscle sarcoglycan-sarcospan complex.. J Biol Chem.

[15] Ramirez-Sánchez I, Rosas-Vargas H, Ceballos-Reyes G, Salamanca F, Coral-Vázquez RM (2005). Expression analysis of the SG-SSPN complex in smooth and endothelial cells of human umbilical cord vessels.. J Vasc Res.

[16] Ito K, Kimura S, Ozasa S, Matsukura M, Ikezawa M (2006). Smooth muscle-specific dystrophin expression improves aberrant vasoregulation in mdx mice.. Hum Mol Genet.

[17] Dye WW, Gleason RL, Wilson E, Humphrey JD (2007). Altered biomechanical properties of carotid arteries in two mouse models of muscular dystrophy.. J Appl Physiol.

[18] Coral-Vazquez R, Cohn RD, Moore SA, Hill JA, Weiss RM (1999). Disruption of the sarcoglycan-sarcospan complex in vascular smooth muscle: a novel mechanism for cardiomyopathy and muscular dystrophy.. Cell.

[19] Durbeej M, Cohn RD, Hrstka RF, Moore SA, Allamand V (2000). Disruption of the β-sarcoglycan gene reveals pathogenetic complexity of limb-girdle muscular dystrophy type 2E.. Mol Cell.

[20] Wheeler MT, Allikian MJ, Heydemann A, Hadhazy M, Zarnegar S (2004). Smooth muscle cell-extrinsic vascular spasm arises from cardiomyocte degeneration in sarcoglycan-deficient cardiomyopathy.. J Clin Invest.

[21] Yoshida T, Owens GK (2005). Molecular determinants of vascular smooth muscle cell diversity.. Circ Res.

[22] Rzucidlo EM, Martin KA, Powell RJ (2007). Regulation of vascular smooth muscle cell differentiation.. J Vasc Surg.

[23] Fritz KE, Jarmolych J, Daoud AS (1970). Association of DNA synthesis and apparent dedifferentiation of aortic smooth muscle cells in vitro.. Exp Mol Pathol.

[24] Thyberg J (1996). Differentiated properties and proliferation of arterial smooth muscle cells in culture.. Int Rev Cytol.

[25] Kokubo T, Uchida H, Choi ET (2007). Integrin αvβ3 as a target in the prevention of neointimal hyperplasia.. J Vasc Surg.

[26] Ström Å, Wigren M, Hultgårdh-Nilsson A, Saxena A, Gomez MF (2007). Involvement of the CD1d natural killer T cell pathway in neointima fotmation after vascular injury.. Circ Res.

[27] Ström Å, Nordin Fredriksson G, Schiopu A, Ljungcrantz I, Söderberg I (2007). Inhibition of injury-induced arterial remodeling and carotid atherosclerosis by recombinant human antibodies against aldehyde-modified apoB-100.. Atherosclerosis.

[28] Schuler F, Sorokin L (1995). Expression of laminin isoforms in mouse myogenic cells in vitro and in vivo.. J Cell Sci.

[29] Gawlik K, Miyagoe-Suzuki Y, Ekblom P, Takeda S, Durbeej M (2004). Laminin α1 chain reduces muscular dystrophy in laminin α2 chain deficient mice.. Hum Mol Genet.

[30] Inoue T, Node K (2009). Molecular basis of restenosis and novel issues of drug-eluting stents.. Circ J.

[31] Ström Å, Wigren M, Hultgårdh-Nilsson A, Saxena A, Gomez MF (2007). Involvement of the CD1d-natural killer T cell pathway in neointima formation after vascular injury.. Circ Res.

[32] Pöling J, Szibor M, Schimanski S, Ingelmann M-E, Rees W (2011). Induction of smooth muscle cell migration during arteriogenesis is mediated by Rap2.. Arterioscler Thromb Vasc Biol.

[33] Hou R, Liu L, Anees S, Hiroyasu S, Sibinga NES (2006). The Fat1 cadherin integrates vascular smooth muscle cell growth and migration signals.. J Cell Biol.

[34] Zahradka P, Wright B, Fuerst M, Yurkova N, Molnar K (2006). Peroxisome proliferator-activated receptor α and γ ligands differentially affect smooth muscle cell proliferation and migration.. J Pharmacology & Exp Therapeutics.

[35] Gawlik K, Durbeej M (2011). Skeletal muscle laminin and MDC1A: pathogenesis and treatment strategies.. Skeletal Muscle.

[36] Cohn RD, Henry MD, Michele DE, Barresi R, Saito F (2002). Disruption of Dag1 in differentiated skeletal muscle reveals a role for dystroglycan in muscle regeneration.. Cell.

[37] Dingemans KP, Teeling P, Lagendijk JH, Becker AE (2000). Extracellular matrix of the human aortic media: an ultrastructural histochemical and immunohistochemical study of the adult aortic media.. Anat Rec.

[38] Rauch U, Saxena A, Lorokowski S, Rauterberg J, Björkbacka H (2011). Laminin isoforms in atherosclerotic arteries from mice and man.. Histol Histopathol.

[39] Finn AV, Nakano M, Narula J, Kolodgie FD, Virmani R (2010). Concept of vulnerable/unstable plaque.. Arterioscler Thromb Vasc Biol.

[40] Tran PK, Tran-Lundmark K, Soininen R, Tryggvason K, Thyberg J (2004). Increased intimal hyperplasia and smooth muscle cell proliferation in transgenic mice with heparin sulfate-deficient perlecan.. Circ Res.

[41] Quignard JF, Harricane MC, Ménard C, Lory P, Nargeot J (2001). Transient down-regulation of L-type Ca2+ channel and dystrophin expression after balloon injury in rat aortic cells.. Cardiovasc Res.

