# Supplementary figures and images for: Increased Neointimal Thickening in Dystrophin-Deficient mdx Mice
Source: PLoS One. 2012 Jan 4;7(1):e29904. doi: 10.1371/journal.pone.0029904 (PMC3251593; doi:10.1371/journal.pone.0029904)

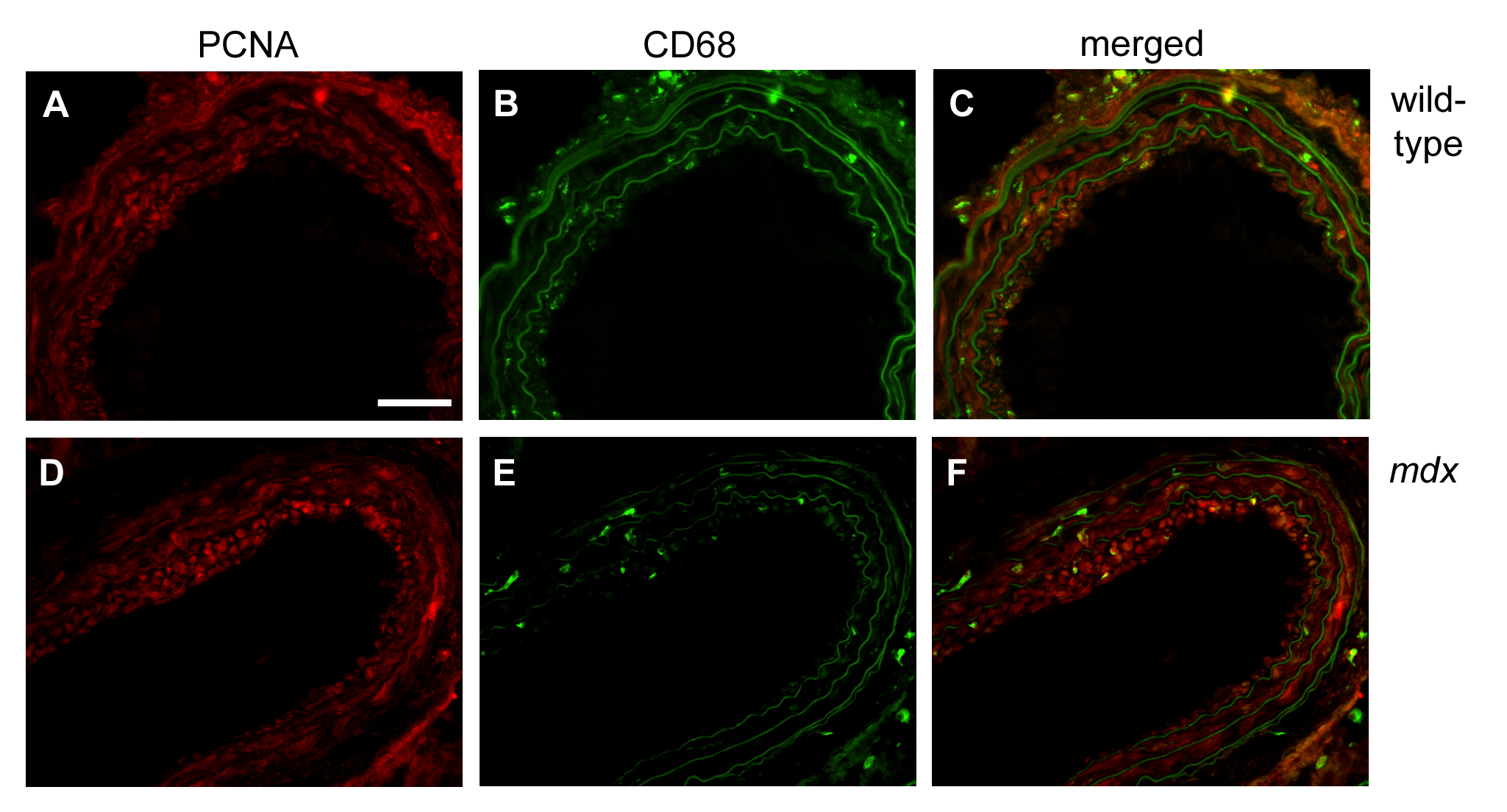

Supplement: Figure S1 — Immunofluorescence staining of PCNA and CD68 positive macrophages in injured carotid arteries. Sections of carotid lesions from wild-type (A, B, C) and mdx (D, E, F) mice were stained for PCNA (A and D) and CD68 (B and E). Images from these staining were merged in C and F showing that PCNA-positive cells were located in the neointima where few CD68-positive cells were found. Bar = 50 µm. The elastic laminae are shown by autofluorescence in the green channel. (TIFF) [file pone.0029904.s001.tiff]

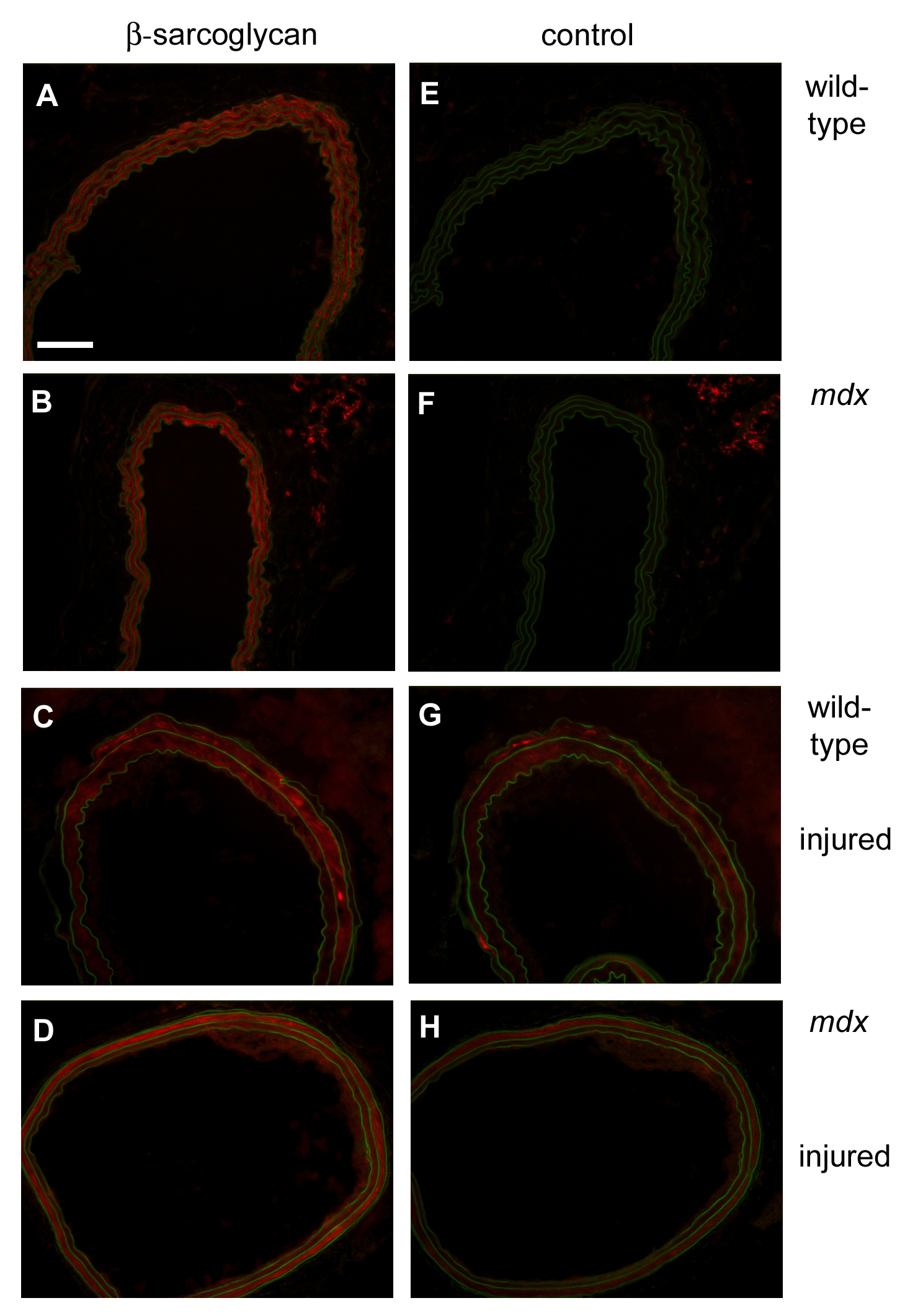

Supplement: Figure S2 — Immunohistochemical staining of β-sarcoglycan in uninjured and injured carotid arteries. Carotid arteries of uninjured wild-type (A, E) and mdx (B, F) mice and of injured wild-type (C, G) and mdx (D, H) were stained with a primary antibody against β-sarcoglycan (A–D) or without (E–H, control for anti-mouse IgG) (in red). Autofluorescence, in particular of the elastin layers of the media, is presented in green. Note the background staining of the secondary anti-mouse IgGs in the control of the injured, but not uninjured media. Bar = 100 µm. (TIFF) [file pone.0029904.s002.tiff]
